# Drug Resistance of Pseudomonas aeruginosa and Enterobacter cloacae Isolated from ICU, Babol, Northern Iran

**Published:** 2013

**Authors:** Masoomeh Bayani, Sepideh Siadati, Ramzan Rajabnia, Ali Asghar Taher

**Affiliations:** 1 *Infectious Disease and Tropical Research Center, Babol University of Medical Sciences, Babol, Iran.*; 2 *Department of Pathology, Babol University of Medical Sciences, Babol, Iran.*

**Keywords:** *Intensive care unit*, *nosocomial infections*, *P aeruginos*, *E cloacae*

## Abstract

Multidrug resistant (MDR) bacteria are spread throughout the world which causes nosocomial infections, especially in Intensive Care Unit (ICU). This study aimed to investigate the resistance pattern of *Pseudomonas aeruginosa* and* Enterobacter cloacae* isolated from patients in the ICU. During 2011-2012, 30 isolates for each *P. aeruginosa *and* E. cloacae* were collected from the patients who acquired nosocomial infection after admition to the ICU at the hospitals affiliated to Babol University of Medical Sciences, Babol, northern Iran. Antimicrobial susceptibility test was performed for five category antibiotics by microdilution method. The data were analyzed by SPSS version 20 and p<0.05 was considered statistically significant. The highest resistance rate of* P. aeruginosa* was seen to amikacin (53.3%) followed by ceftazidime (43.3%). Also, 16.7% of *E. cloacae* was resistant to ceftazidime. Among *P. aeruginosa* isolates,18 (60%) were MDR while no *E. cloacae* isolates were MDR. The significant correlation was only demonstrated between MDR* P. aeruginosa* and the reason of hospitalization (P=0.004). In conclusion, there was alarming amount of* P. aeruginosa *MDR in patients in the ICU which could lead to a hazardous outcome for the patients. Therefore, new prevention policies regarding to hospital infection should be established. Also, the periodical assessment of bacterial resistance pattern particularly in ICUs should be performed.

Hospital-acquired infections (HALs), also called nosocomial infections are associated with an increase in morbidity, mortality and health-care costs (1). Patients requiring intensive care unit (ICU) are prone to HALs 5 to 7-fold compared on general hospital wards (2-3). Gram-negative bacilli are prevalent cause of these infections, with 20% to 30% mortality rate, and *Pseudomonas aeruginosa* (*P. aeruginosa*) is the most common agent (4-5). Also, the prevalence of multidrug resistant (MDR) bacteria has increased all over the world (5-7). The mechanism of resistance of gram-negative bacteria results from mutation of genes and transmissible genetic elements with high dissemination potential (transposon or integrin). They can spread rapidly among bacteria. The severe outcome and high morbidity and mortality due to these bacterial infections emphasize the prompt need for obtaining data along with the resistance pattern that are benefical in guiding physicians for appropriate antibiotic therapy, decreasing the length of stay of patients in ICU, as well as decreasing the mortality, morbidity and health cost. Also, these data are of great value to make health sterategies and prog-rams. There are a few available data concerning antibiotic resistance from ICU in Iran (5, 8-9). On the other hand, resistance pattern are so varied among different countries and even different regions of the same country. This study aimed to investigate the resistance pattern of *P. aeruginosa* and *E. cloacae* isolated from patients admitted to the ICU of hospital affiliated to Babol University of Medical Sciences, Babol.northern Iran.

## Materials and Methods

**Bacterial isolates**

This cross-sectional prospective study was conducted in Hospitals affiliated to Babol University of Medical Sciences from 2011 to 2012. Urine and sputum specimens were collected from the patients after 48 hours, following admission to ICUs. All patients with recent history of infectious diseases or symptoms of infection at admission were excluded.

In order to obtain isolated colonies, all specimens were cultured on nutrient agar (Merk, Germany) using strike plate method and then incubated at 37°^c^ for 18 to 24 hours. To isolate *P. aeruginosa *and* E. cloacae*, the purred colonies from any specimens were cultured on blood agar, choclate agar and EMB agar. Gram staining and standard biochemical tests were also processed for further identification of the bacteria (9). The bacterial culture yielding greater than 10^5^cfu/ml had been considered as a positive culture.

**Antimicrobial Susceptibility Testing**

Susceptibility test was carried out using microdilution method according to Clinical and Laboratory Standard Institute (CLSI 2010 M02-A9) guidelines (4, 8). Five antibiotics groups were used as described below: group: penicillin and ß -lactamase inhibitor [ampicillin / salbactam (AST) and piperacillin / tazobactam (PTZ)]; group 2: quinolone [ciprofloxacin (CRO)]; group 3: aminoglycosid [amikacin (AMK)]; group 4 carbapenem [imipenem (IMP)] and group 5: 3rd and the 4th generation cephalosporins [ceftazidime (CAZ) and cefepime (CPM)] (Merck, Germany), were used.

Minimum inhibitory concentration (MIC) of these antimicrobial agents were interpreted and classified as susceptible, intermediate and resistant. Based on the available data, MDR was reported as resistant to at least 3 groups of antibiotics including: 1) ampicillin / salbactam or piperacillin / tazobactam 2) ciprofloxacin 3) amikacin 4) imipenem 5) cefepime or ceftazidime (10). Additional data including age, gender, duration of stay, type of specimen (urine or sputum) and the reason of admission (surgical or nonsurgical) were collected through questionnaires.

**Statistical Analysis**

The SPSS software version 20 (SPSS Inc, Chicago, IL) was used to analye the data. A p-value of <0.05 was considered statistically significant.

## Results

The results obtained from susceptibility test using *P. aeruginosa *and* E. cloacae* were shown in table1. These findings indicated that the resistant rate of *P. aeruginosa *to amikacin, ceftazidim*, *cefepime and imipenem, and ciprofloxacin were 53.3%, 43.3%, 40% and 33.3%, respectively. 26.6% and 50% of *P. aeruginosa *isolate were resistance and intermediate resistance to Pipera-cillin-tazobactam, respectively. The isolated *E. cloacae* were more sensitive to the antibiotics used in the current study. The resistant rate of* E. cloacae* to ceftazidim*, *cefepime, imipenem, and ciprofloxacin were 16.7%, 13.3%, 6.7% and 6.7%, in that order. The most effective antibiotics were ciprofloxacin and amikacin for *P. aeruginosa *and* E. cloacae*, respectively.

18 out of 30 isolates of *P. aeruginosa* (60%) were MDR and 5 out of 18 MDR isolates (27. 7%) were resistant to 4 antimicrobial groups (Table 2). Furthermore, MDR isolates of *E. cloacae* were not found. The results obtained from testing any association between MDR isolates of *P. aeruginosa *and different variables such as age of patients, type of specimen demonstrated that there is a significant correlation between MDR isolates and the reason of hospitalization (P=0.004) (Table 3).

## Discussion

Emerging of HAIs and MDR pathogens is one of the important subject around the world and great concern should be paid to it. Indeed, the resistance of bacteria to antimicrobial agents differs by country and region, indicating the need to conduct regional studies. The results obtained from the current study showed the *P. aeruginosa* isolates were more resistant to various antibacterial agents in comparison to *E. cloacae* isolates (Table 1).

**Table 1:** Antibiotic susceptibility patterns of *P. aeruginosa* and *E. cloacae* isolated from ICU patients

***E. cloacae***			***P. aeruginosa***			**Antibiotics**
Resistance	Intermediate	Sensitive	Resistance	Intermediate	Sensitive	
2(%6.7)	4(%13.3)	24(%80)	16(%53.3)	5(%16.6)	9(%30)	Ampicilin-salbactam
3(%10)	16(%53.3)	11(%36.7)	8(%26.6)	15(%50)	7(%23.3)	Piperacillin-tazobactam
2(%6.7)	6(%20)	22(%73.3)	10(%33.3)	3(%10)	17(%56.6)	Ciprofloxacin
2(%6.7)	0(%0)	28(%93.3)	16(%53.3)	0(%0)	14(%46.6)	Amikacin
2(%6.7)	5(%16.7)	23(%76.7)	12(%40)	8(%26.6)	10(%33.3)	Imipenem
4(%13.3)	5(%16.7)	21(%70)	12(%40)	9(%30)	9(%30)	Cefepime
5(%16.7)	4(%13.3)	21(%70)	13(%43.3)	10(%33.3)	7(%23.3)	Ceftazidime

**Table 2:** MDR pattern of *P. aeruginosa*

MDR *P. aeruginosa* isolates																																				Antimicrobial agent		Antimicrobial category	
18		17		16		15		14		13		12		11		10		9		8		7		6		5		4		3		2		1					
x						x		x		x		x		x		x						x				x				x				x		Amikacin		Aminoglycosides	
		x		x				x		x		x						x		x		x						x		x		x				Imipenem		Antipseudomonal carbapenems	
x		x		x				x		x		x				x		x				x		x		x				x		x		x		Cefepime		Antipseudomonal cephalosporins	
						x				x				x						x								x		x		x		x		Ceftazidime			
		x						x		x		x		x				x						x		x										Ciprofloxacin		Antipseudomonal fluoroquinolones	
x		x		x		x				x						x		x		x				x				x		x				x		Piperacillin-tazobactam		Antipseudomonal penicillins + β-lactamase inhibitors	
				x		x						x		x				x		x		x		x		x		x		x				x		Ampicilin-salbactam			

**Table 3:** Demographic parameters according to MDR * P. aeruginosa* isolated from ICU patients

P-Value	MDR N=18	Susceptible N=12	Variables
0.70	62±20.7	59.62±22.5	Age(Mean + SD)
0.58			Gender
	7(%38.9)	5(%41.6)	Male
	11(%61.1)	7(%58.3)	Female
0.945	49.8±42	50.7±45.8	Length of stay(Mean + SD)
0.41			Type of specimen
	10(%55.5)	8(%66.6)	Urine
	8(%44.5)	4(%33.4)	Sputum
0.25			Hospital
	15(%83)	15(%40)	Rohani
	1 (%5.6)	19(%45)	Shahid Beheshti
	2(%11)	6(%14)	Yahyanejad
0.004			Reason of stay
	2(%11)	8(%66.6)	surgical
	16(%89)	4(%33.4)	Nonsurgical

It also showed that the prevalence of *P. aeruginosa* resistant isolates was increased. For example, the rate of *P. aeruginosa * resistance to amikacin, the most effective antimicrobial agent, was 53.3% which was greater than the results obtained from a recent study in ICU patients indicating that 46.2% of the isolates were resistant to amikacin (11). These results were also supported by several studies (12-13). But these results are in contrast with a study from India that demonstrated 71% of the isolates were resistant to these antibiotics (14). Also, the rate of *E. cloacae* resistance to amikacin were so different in several studies that ranged from 36% to 100% (13, 15). We showed that 7% of *E. cloacae* were resistant which was similar to the study from Belgium (16).

Furthermore, the resistance rates of the* P. aeruginosa *and* E. cloacae* isolates to ciprofloxacin were 33.3% and 7%, respectively. This finding is in agreement with other studies reporting 33% and 38.9% of the bacteria were resistat to this antibiotic (11, 15). However, other studies reported that the resistance rate of *P. aeruginosa* isolates ranged from 4-79% (17-21). There are several studies from Iran reporting the higher resistance rate of *E. cloacae* to ciprofloxacin (9, 13, 15).

Also, the resistance rate of the* P. aeruginosa* to piperacillin / tazobactam was 26.6% which was relatively similar to the study performed by Japoni et al. from Shiraz, Iran (25%) and another study from Tehran, Iran (33%). The resistance of the *E. cloacae* isolates was 10% which was much lower than the reports from Shiraz (47%) and Tehran (28%) (9, 15).

Moreover, imipenem is another effective antimicrobial agent used for treatment of *P. aeruginosa* and* E. cloacae* infections and there are several studies reporting that the resistance rate of the bacteria to these antibiotic have increased throughout the world (12). We found that 40 % and 7% of* P. aeruginosa* and* E. cloacae* isolates were resistant to imipenem, respectively, which was close to some reports from Iran(9, 15). However, these results were different from others (16, 22). The same result was obtained for *P. aeruginosa* to cefepime and ceftazidime (40%) but the resistance rate of the *E. cloacae* isolates was higher (13.3% and 16.7%) (13, 15, 19, 23, 24).

In the current study, 53.3% and 7% of the isolated *P. aeruginosa* and *E. cloacae* were resistant to ampicillin / salbactam. There are several studies that reported the higher rate of resistance (4, 14, 16).

In addition, the current study demonstrated that 60% of the *P. aeruginosa* isolates were MDR while none of the *E. cloacae* isolates were MDR. This rate was higher than the results obtained from other studies from Iran (42. 3%) or another report showed that the prevalence of MDR-* P. aeruginosa* increased in the USA and 16% of the isolates were resistant to three or more of the core drugs (12, 25). However, the present study found that a significant correlation between the reason of hospitalization (surgical and nonsurgical) and MDR-* P. aeruginosa* (p=0.004) but no association was seen between age, gender, type of specimen, length of hospitalization and different hospitals with MDR-* P. aeruginosa*. These findings are similar to several studies(6, 26-27).

However, possible explanations for the differences between our results with the findings of other studies are dissimilarity of antibiotics consumption, national and international antibiotics policy and hygiene measurement in different regions. In fact, the prevalence and resistance pattern of infectious agents are varied among the different hospitals in the same area or different regions throughout the world (12).

Although this study had valuable results particularly it performed in three different hospitals, but it suffered from some limitations. First, the number of isolates was scanty. Second, it only considered two pathogens. In conclusion, considering the high incidence* of P. aeruginosa* MDR in ICU could lead to a hazardous outcome for the patients. Therefore, new prevention policies regarding hospital infections should be established. Also, the periodical assessment of bacterial resistance pattern particularly in ICUs should be performed.
